# A High-Throughput Fluorescence Polarization-Based Assay for the SH2 Domain of STAT4

**DOI:** 10.3390/mps5060093

**Published:** 2022-11-23

**Authors:** Angela Berg, Martin Gräber, Sebastian Schmutzler, Ralf Hoffmann, Thorsten Berg

**Affiliations:** 1 Institute of Organic Chemistry, Leipzig University, Johannisallee 29, 04103 Leipzig, Germany; 2 Max Planck Institute of Biochemistry, Department of Molecular Biology, Munich Center for Integrated Protein Science (CIPSM), Am Klopferspitz 18, 82152 Martinsried, Germany; 3 Institute of Bioanalytical Chemistry and Center for Biotechnology and Biomedicine, Leipzig University, Deutscher Platz 5, 04103 Leipzig, Germany

**Keywords:** assay development, fluorescence polarization, protein-protein interactions, SH2 domain, STAT4

## Abstract

The signal transducer and activation of transcription (STAT) proteins are a family of Src homology 2 (SH2) domain-containing transcription factors. The family member STAT4 is a mediator of IL-12 signalling and has been implicated in the pathogenesis of multiple autoimmune diseases. The activity of STAT4 requires binding of phosphotyrosine-containing motifs to its SH2 domain. Selective inhibitors of the STAT4 SH2 domain have not been published to date. Here, we present a fluorescence polarization-based assay for the identification of inhibitors of the STAT4 SH2 domain. The assay is based on the interaction between the STAT4 SH2 domain and the fluorophore-labelled peptide 5-carboxyfluorescein-GpYLPQNID (K_d_ = 34 ± 4 nM). The assay is stable with respect to DMSO concentrations of up to 10% and incubation times of at least 8 h. The Z’-value of 0.85 ± 0.01 indicates that the assay is suited for use in high-throughput screening campaigns aimed at identifying new therapeutic modalities for the treatment of autoimmune diseases.

## 1. Introduction

The signal transducer and activation of transcription (STAT) proteins are a family of Src homology 2 (SH2) domain-containing transcription factors [1,2]. STAT proteins bind to phosphotyrosine motifs on the cytoplasmic tails of activated cytokine or growth factor receptors via their SH2 domains. The receptor-bound STAT proteins are then activated by phosphorylation of a conserved tyrosine residue, either by receptor-associated Janus kinases (JAKs), other cytoplasmic tyrosine kinases or intrinsic receptor tyrosine kinase activity. Activated STAT proteins dimerise via reciprocal phosphotyrosine-SH2 domain interactions and translocate to the nucleus, where they regulate transcription of their target genes.

The STAT family member STAT4 is activated by phosphorylation of tyrosine 693 in response to receptor binding of the cytokine IL-12 [3]. The IL-12 receptor is predominantly found on T-cells and natural killer (NK) cells; in both cell types signalling via STAT4 induces the production of IFN-γ, which plays an important role in the upregulation of both innate and adaptive immune processes [4]. STAT4 can also be activated by Type I interferons, IL-2, IL-21, IL-23 and IL-35 [5]. Consistent with its role in the immune response, STAT4 has been shown to be involved in the pathogenesis of autoimmune diseases including inflammatory bowel disease, multiple sclerosis, rheumatoid arthritis and diabetes mellitus [4,5]. The involvement of STAT4 in tumourigenesis is less clear. In certain tumour types, STAT4 signalling has been shown to promote growth, invasiveness and/or metastasis. However, in other forms of cancer STAT4 appears to play a protective role [6,7]. Since the activation and dimerization of STAT proteins requires binding to the SH2 domain, selective small-molecule inhibitors of the STAT4 SH2 domain represent potential new therapeutic agents for the treatment of autoimmune diseases [5] and molecular tools for the investigation of the role of STAT4 in cancer. However, no such inhibitor has been published to date.

Fluorescence polarization (FP) assays can be used to assess binding between two partners of significantly different molecular weights in solution [8,9]. The smaller binding partner, in this case a peptide, is labelled with a fluorophore. When linearly polarized light of the excitation wavelength is applied to the fluorophore, only those molecules with a suitable spatial orientation relative to the plane of polarization are excited. This results in excited fluorophores with a highly uniform spatial orientation. In the time between excitation and fluorescence emission, the high rotational mobility of the fluorophore-labelled peptide results in a substantially less uniform spatial orientation, reflected in a low degree of polarization of the emitted fluorescence. Since the rotational mobility of a molecule correlates with its molecular volume (a rough measure of molecular weight), binding of the labelled peptide to a larger molecule, in this case a protein, leads to an increase in polarization of the emitted fluorescence. Conversely, inhibition of binding between the protein and the fluorophore-labelled peptide by a small molecule inhibitor or unlabelled peptide liberates the fluorophore-labelled peptide from the protein, reducing the degree of fluorescence polarization.

We have previously published FP-based assays for high-throughput screening of the ability of small molecules to inhibit binding of fluorescent-labelled phosphopeptides to the SH2 domains of STAT3 [10], STAT5a [11], and STAT5b [12]. Here, we present a detailed description of a fluorescence polarization-based assay suitable for high-throughput screening for STAT4 SH2 domain inhibitors. The assay has already proved its utility in the specificity analysis of inhibitors found to inhibit other phosphorylation-dependent protein-protein interactions [13,14,15,16,17,18].

## 2. Materials and Methods

### 2.1. Peptide Sequences

Phosphopeptides for direct binding assays were labelled with 5-carboxyfluorescein (CF) at the N-terminus. Unless stated otherwise, the C-termini of the peptides were synthesised as free carboxylic acids. The following peptides were used for direct binding assays: 5-CF-GpYLPSNID, 5-CF-GpYLPQNID, 5-CF-GpYDKPHVL, 5-CF-GpYLPQTV-NH_2_, 5-CF-GpYLVLDKW, 5-CF-GpYVPWQDLI, 5-CF-GpYEEIP, and 5-CF-GPMQSpTPLNG. The unlabelled peptides Ac-GpYLPQNID, Ac-pYLPQTV-NH_2_, DTpYLVLDKWL, GpYDKPHVL, GpYEEIP and MAGPMQSpTPLNGAKK were used in competitive inhibition assays. Peptides were analysed by reversed-phase HPLC and mass spectrometry.

### 2.2. Cloning and Protein Expression

Amino acids 136 to 705 of human STAT4, which represents the coiled coil, DNA-binding, linker and SH2 domains, were amplified from placental cDNA via PCR and cloned via FseI and AscI restriction enzyme sites into a modified pQE70 vector carrying an N-terminal MBP tag and a C-terminal 6×His tag. The point mutant STAT4 R598A was generated by following the QuikChange site-directed mutagenesis protocol (Agilent Genomics). The resulting STAT4 constructs were expressed from Rosetta BL21DE3 cells (Novagen) as previously described for STAT5 constructs [19], and purified via affinity chromatography using His-Bind resin (Merck Millipore). Cloning, expression and purification of murine STAT3 (amino acids 127 to 722, identical to human STAT3 on the protein level) [10] and human STAT6 (amino acids 110 to 651) were previously described [13]. Proteins were dialysed against a buffer containing 100 mM NaCl, 50 mM Hepes pH 7.5, 1 mM EDTA, 1 mM dithiothreitol (DTT), 10% (*v*/*v*) glycerol and 0.1% (*v*/*v*) Nonidet P40 (NP-40) substitute, snap-frozen in liquid nitrogen and stored at −80 °C until use.

### 2.3. Fluorescence Polarization Assays

**Figure 1:**
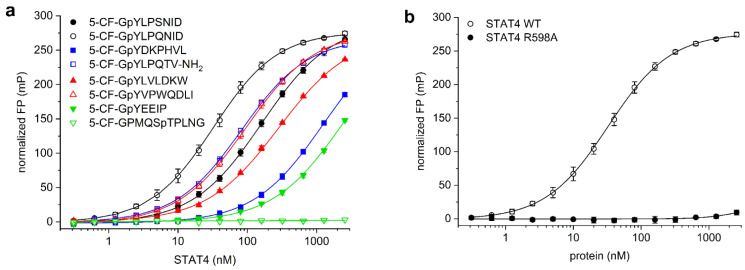
(**a**) Binding of fluorescent-labelled phosphopeptides probes to STAT4. (**b**) Binding of 5-CF-GpYLPQNID to STAT4 wild-type (WT) and the point mutant STAT4 R598A.

**Figure 2:**
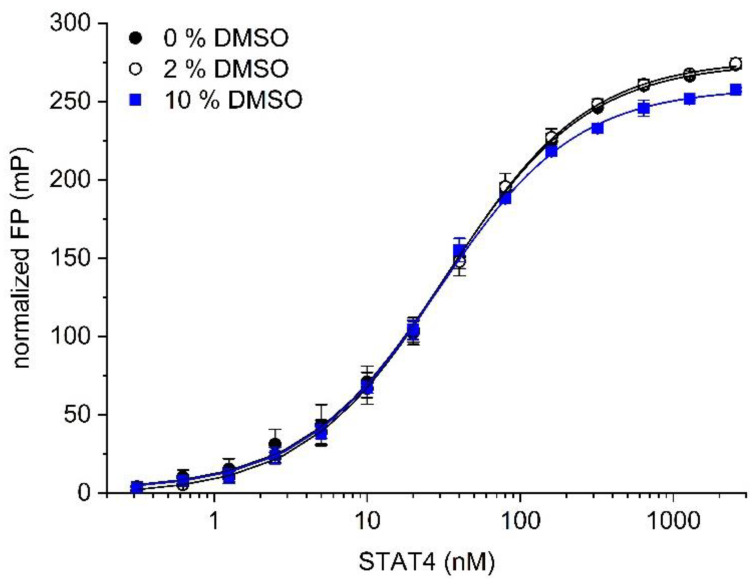
The effect of DMSO on binding of the probe 5-CF-GpYLPQNID to STAT4.

**Figure 3:**
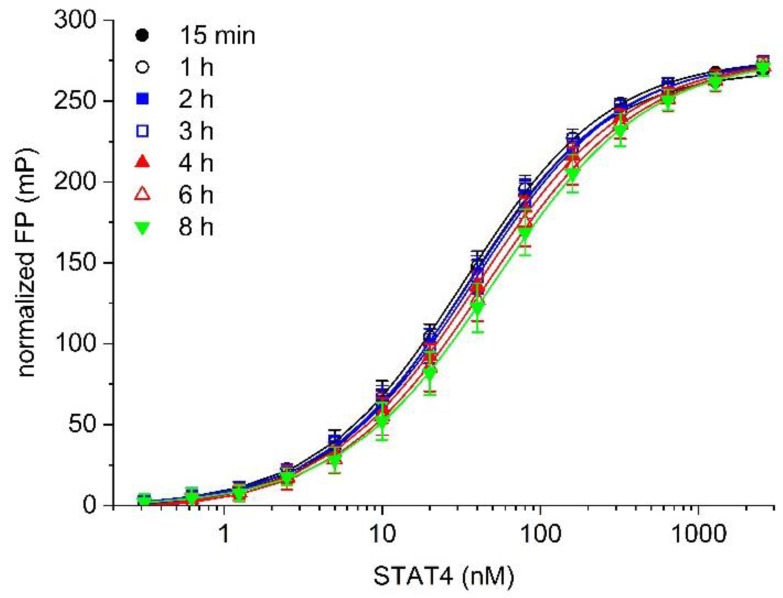
Temporal stability of binding of the probe 5-CF-GpYLPQNID to STAT4.

**Figure 4:**
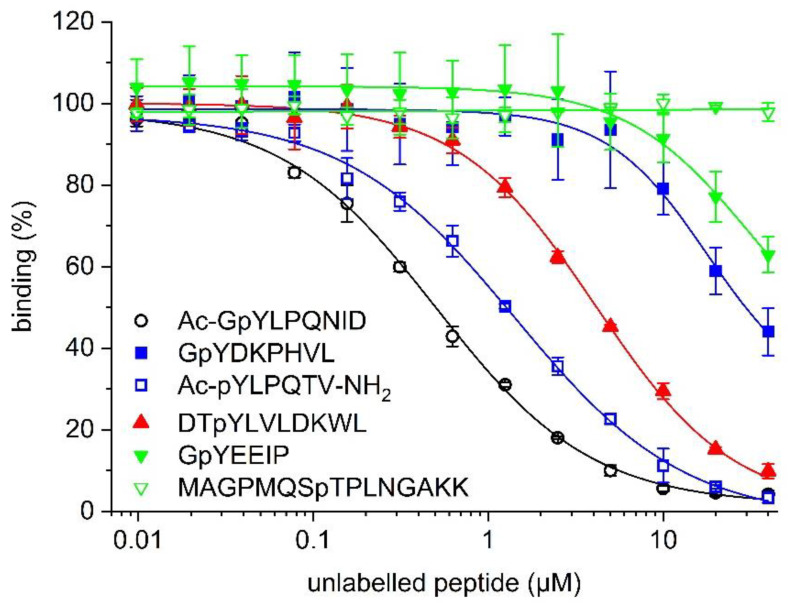
Competitive inhibition of binding between 5-CF-GpYLPQNID and STAT4 (33 nM) by the indicated unlabelled phosphopeptides.

Binding of fluorescent-labelled peptides to the truncated STAT4 protein was analysed using an Infinite F500 plate reader (Tecan) as previously published for STAT5b [12]. Unless otherwise stated, FP assays were performed in a buffer consisting of 10 mM Tris/HCl, 50 mM NaCl, 1 mM EDTA, 0.1% (*v*/*v*) NP-40 substitute, 2% (*v*/*v*) DMSO and 1 mM DTT at a pH of 8.0. All steps were carried out at room temperature. For the binding assays shown in Figure 1, Figure 2 and Figure 3, the prediluted STAT4 protein was incubated for 1 h before addition of the fluorophore-labelled peptide, to mimic the incubation period with test compounds. For the inhibition assay shown in Figure 4, STAT4 was incubated with unlabelled peptide for 1 h before addition of the fluorophore-labelled peptide (final STAT4 concentration: 33 nM). Fluorescence polarization was read 1 h after addition of the labelled peptide, in non-treated black 384-well microplates (Corning). Fluorescent-labelled peptides were used at a final concentration of 10 nM unless otherwise stated. Binding and inhibition curves were plotted using OriginPro 2019 software (OriginLab). Fluorescence polarization values were normalized by subtracting the FP values of the wells containing the fluorophore-labelled peptides only. IC_50_ values represent the concentration at which 50% of the maximal protein binding activity (activity in the absence of inhibitor) was observed. IC_50_ values from three independent experiments were used to calculate an average value and the corresponding standard deviation. IC_50_ values were converted to inhibition constants (K_i_) using the published equation [20], with average values calculated as for IC_50_ values. Affinities and inhibitory activities are given as the mean value ± standard deviation of 3 independent experiments, unless stated otherwise.

### 2.4. Calculation of Z’

The *Z’* value was calculated using the equation *Z’* = 1 − (3 × SD_bound_ + 3 × SD_free_)/(mP_bound_ − mP_free_), where SD is standard deviation and mP is fluorescence polarization [21]. The “free” state is represented by 10 nM of the fluorescent-labelled peptide 5-CF-GpYLPQNID in the absence of protein; the “bound” state is represented by 10 nM of 5-CF-GpYLPQNID in the presence of 33 nM STAT4 protein. Fluorescence polarization was measured after 1 h of incubation. Three independent experiments were performed, with 119 wells for each condition per experiment.

## 3. Results and Discussion

STAT4 binds to the activated IL-12 receptor via a motif including tyrosine 800 in the receptor tail, and also to the corresponding peptide THDGpYLPSNIDD [22]. The related peptide sequence SHEGpYLPSNID was shown to bind to STAT4 with high affinity, and binding was improved by exchanging the serine in the pY + 3 position for glutamine, giving SHEGpYLPQNID [23]. Since amino acids N-terminal of the phosphorylated tyrosine residue do not directly bind to STAT SH2 domains [24,25], and conformational flexibility between the fluorophore and the core binding motif reduces the assay window of fluorescence polarization assays, the fluorophore was attached at the N-terminus of the glycine residue. The use of a glycine spacer between the fluorophore and phosphotyrosine avoids negative interference of the fluorophore with the SH2 domain [26], and has previously been successfully used in the design of probes for STAT1 [26], STAT3 [10], STAT5a/b [11,12], and STAT6 [26]. We therefore assessed binding of the 5-carboxyfluorescein (CF)-labelled peptides 5-CF-GpYLPSNID and 5-CF-GpYLPQNID to a truncated STAT4 protein containing the SH2 domain. The glutamine-containing peptide 5-CF-GpYLPQNID (K_d_ = 34 ± 4 nM, Figure 1a) had a significantly higher affinity for STAT4 than 5-CF-GpYLPSNID (K_d_ = 160 ± 6 nM). In consequence, 5-CF-GpYLPQNID was selected for further use in STAT4 assay development.

While binding of SH2 domains to peptide motifs is crucially dependent on the presence of a phosphorylated tyrosine residue, SH2 domains show some overlap in their preferred binding sequences with respect to the amino acids directly C-terminal of the phosphotyrosine, leading to promiscuity in SH2 domain binding [27]. In order to assess the selectivity of STAT4 for binding to 5-CF-GpYLPQNID, we also analysed STAT4 binding to the corresponding optimal fluorescein-labelled peptides for other members of the STAT family. The peptide sequence used in the STAT3 assay (5-CF-GpYLPQTV-NH_2_) [10], which is derived from the gp130 subunit of the IL-6 receptor and which shares the core binding motif pYLPQ of the STAT4 probe, displayed 2.4-fold lower affinity for STAT4 (K_d_ = 82 ± 2 nM, Figure 1a) than the preferred STAT4-binding peptide 5-CF-GpYLPQNID. Similarly, the STAT4 probe 5-CF-GpYLPQNID has fourfold weaker affinity for STAT3 (K_d_ = 131 ± 6 nM, Figure S1 in the Supplementary Material) than for STAT4 (K_d_ = 34 ± 4 nM).

The STAT6-binding peptide 5-CF-GpYVPWQDLI [13,23] showed a 3-fold lower affinity for STAT4 (K_d_ = 93 ± 8 nM, Figure 1a) than the STAT4-binding peptide 5-CF-GpYLPQNID (K_d_ = 34 ± 4 nM), indicating that the presence of the bulky tryptophan residue in the pY + 3 position of 5-CF-GpYVPWQDLI is tolerated by STAT4. This is consistent with a literature report stating that the peptide SHEGpYLPWNID, containing tryptophan in the pY + 3 position, is only 2-3-fold less potent against STAT4 (IC_50_ = 0.93 µM) in an ELISA than the peptide SHEGpYLPQNID (IC_50_ = 0.39 µM) with glutamine in the pY + 3 position [23]. In contrast, the STAT4 probe 5-CF-GpYLPQNID, with glutamine in the pY + 3 position, has poor affinity for STAT6 (K_d_ > 2560 nM, Figure S1), suggesting that high affinity for STAT6 requires the presence of tryptophan in this position. This finding is consistent with the literature, which reports a strong preference of STAT6 for tryptophan over glutamine in the pY + 3 position [23].

The STAT5a/b binding peptide 5-CF-GpYLVLDKW [11,12,14], which is derived from the erythropoietin (EPO) receptor, showed an approximately 8-fold weaker affinity for STAT4 (K_d_ = 267 ± 6 nM, Figure 1a) than the preferred STAT4-binding peptide 5-CF-GpYLPQNID. An even lower affinity for STAT4 was observed with the STAT1-binding peptide 5-CF-GpYDKPHVL, derived from the IFN-γ receptor [26] (K_d_ = 1230 ± 284 nM), and with the Src/Lck SH2 domain-binding peptide 5-CF-GpYEEIP [28,29] (K_d_ > 2560 nM). The phosphothreonine-containing peptide 5-CF-GPMQSpTPLNG, which binds to the polo-box domain of Plk1 [30], did not bind to STAT4, demonstrating that the presence of 5-carboxyfluorescein at the N-terminus of a non-binding peptide sequence does not induce unspecific STAT4 binding. In order to confirm a specific interaction between the probe 5-CF-GpYLPQNID and the STAT4 SH2 domain, which is predicted to engage in electrostatic interactions with the phosphate group of phosphotyrosine-containing peptides [24,31,32], we generated the point mutant STAT4 R598A. In this mutant, the conserved arginine residue at the bottom of the phosphotyrosine binding pocket was mutated to alanine. Binding of 5-CF-GpYLPQNID to STAT4 R598A was almost completely abolished (Figure 1b), supporting the notion that the affinity of the probe for wild-type STAT4 is based on selective recognition by the SH2 domain. These data reflect the partially overlapping binding preferences within the STAT family, together with the dependence of SH2 domains on the presence of a phosphotyrosine within the binding motif [33], and confirm selective recognition of STAT4 by the probe 5-CF-GpYLPQNID.

The binding experiments outlined above were carried out using fluorescent-labelled peptides at a concentration of 10 nM. It is important that the concentration of the labelled probe remains significantly below the K_d_ value of the binding curve, since a concentration above the true K_d_ value results in a higher apparent K_d_. Reduction of the concentration of the probe 5-CF-GpYLPQNID to 5 nM or 2 nM gave K_d_ values of 30 ± 1 nM and 30 ± 3 nM, respectively, as compared with 34 ± 4 nM using 10 nM probe. Given the lack of a significant difference between the values, we chose 10 nM to allow for better comparability with the results from FP assays against other proteins used in our laboratory, all of which use 10 nM of tracer. While lower concentrations of tracer would reduce its consumption in high-throughput campaigns, they also reduce the fluorescence readings relative to buffer, and thereby render the assay more susceptible to artefacts originating from autofluorescent test compounds in screening libraries.

Since test compounds for high-throughput screening are typically dissolved in dimethyl sulfoxide (DMSO), the assays outlined above were carried out in the presence of 2% (*v*/*v*) DMSO. Omitting DMSO from the buffer did not affect binding between the probe 5-CF-GpYLPQNID and STAT4 (K_d_ = 34 ± 3 nM, Figure 2), as compared to 34 ± 4 nM in the presence of 2% DMSO. In the presence of 10% (*v*/*v*) DMSO, the K_d_ was only slightly altered (K_d_ = 29 ± 1 nM, Figure 2), indicating that the assay is stable against DMSO at concentrations of up to 10%.

Temporal stability is an important feature of a robust assay. Binding between 5-CF-GpYLPQNID and STAT4 was stable with respect to time, with only a minor loss of binding being observed up to 4 h after addition of the labelled probe, and no more than 35% reduction in affinity by 8 h (K_d_ value 15 min: 33 ± 3 nM; 1 h: 34 ± 4 nM; 2 h: 35 ± 5 nM; 3 h: 38 ± 4 nM; 4 h: 40 ± 6 nM; 6 h: 45 ± 8 nM; 8 h: 51 ± 9 nM, Figure 3). This is advantageous as a practical consideration when designing high-throughput protocols.

In order to confirm the suitability of the STAT4 binding assay for use in competitive inhibition assays, the effect of increasing concentrations of the unlabelled peptide Ac-GpYLPQNID on binding between 5-CF-GpYLPQNID and STAT4 was assessed. Ac-GpYLPQNID inhibited the interaction between STAT4 and the fluorophore-labelled peptide with an IC_50_ value of 0.49 ± 0.04 µM (K_i_ = 0.22 ± 0.02 µM, Figure 4, Table 1). The reversibility of the interaction between 5-CF-GpYLPQNID and STAT4 is indicated by the near-complete (96%) inhibition at 40 µM Ac-GpYLPQNID, with an extrapolated maximum inhibition of 98.4 ± 0.9% at infinite peptide concentrations. The STAT3 inhibitor peptide Ac-pYLPQTV-NH_2_ [10,34] was 2.6-fold less active (IC_50_ = 1.27 ± 0.10 µM, K_i_ = 0.60 ± 0.05 µM). Weaker activities were observed for the STAT5 inhibitor peptide DTpYLVLDKWL [12] (IC_50_ = 4.17 ± 0.13 µM, K_i_ = 1.99 ± 0.06 µM) and the STAT1 inhibitor peptide GpYDKPHVL [26] (IC_50_ = 31.4 ± 6.7 µM, K_i_ = 15.1 ± 3.2 µM). The unlabelled peptide GpYEEIP, which binds to SH2 domains of Src [28] and Lck [29], inhibited STAT4 only to a lesser extent (37 ± 4% inhibition at 40 µM, the highest concentration tested). The Plk1-binding peptide MAGPMQSpTPLNGAKK [30] had no detectable inhibitory effect. Thus, the profile of inhibitory activities of the unlabelled peptides in the competitive inhibition assay (Figure 4, Table 1) closely correlates with the relative binding affinities of the corresponding fluorescein-labelled peptides in the direct binding assay (Figure 1a), indicating that the presence of the fluorophore does not alter the selectivity profile of the underlying peptide sequences.

The K_i_ value of the unlabelled peptide Ac-GpYLPQNID (K_i_ = 0.22 ± 0.02 µM, Table 1) is higher than the K_d_ value of the corresponding fluorophore-labelled peptide 5-CF-GpYLPQNID (K_d_ = 34 ± 4 nM, Figure 1). Characterization of known small-molecule inhibitors of the STAT4 SH2 domain in the FP assay would be required to assess whether this translates into lower activity levels of small-molecule inhibitors in the FP assay as compared to other assay types. Unfortunately, selective small-molecule inhibitors of STAT4 by which to explore this question have not yet been reported. Of note, higher K_i_ values of unlabelled peptides as compared to K_d_ values of the corresponding fluorophore-labelled peptides have also been reported for FP assays against the XIAP BIR3 domain (K_i_/K_d_ = 4) [20] and the Plk1 PBD (K_i_/K_d_ = 5) [30]. These assays have been successfully used to identify selective inhibitors of their target proteins by screening campaigns [35,36,37] and medicinal chemistry efforts [38,39,40,41,42,43,44], suggesting that the (K_i_/K_d_) ratio of the STAT4 assay should not negatively affect its utility.

**Figure 5:**
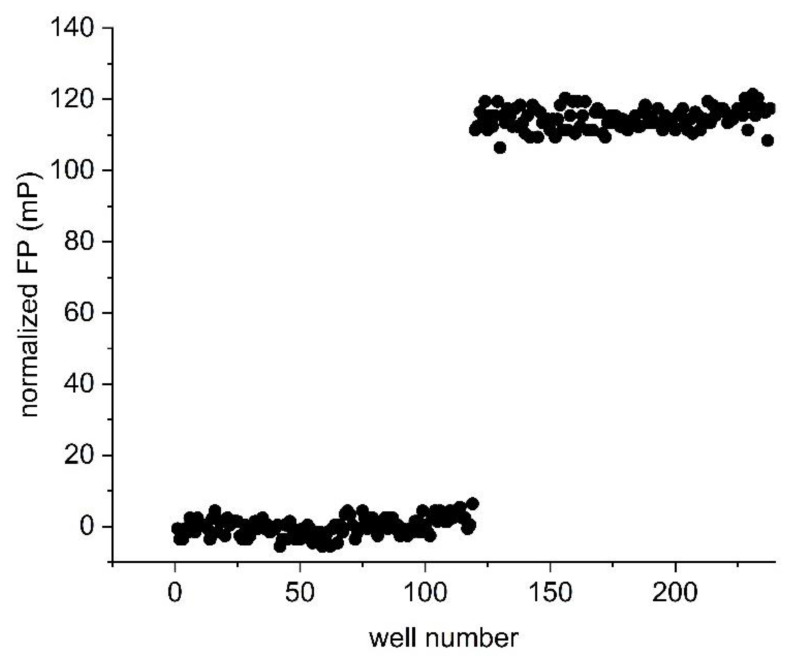
Determination of the Z’ value. Fluorescence polarization was measured in samples containing 10 nM 5-CF-GpYLPQNID in the absence (wells 1–119) and presence (wells 120–238) of 33 nM STAT4. The data were normalized by subtracting the average FP reading of the peptide-only wells from all well FP values. The Z’ value of the representative experiment shown is 0.86.

An assay is only considered to be suitable for high-throughput screening applications if well-to-well variations are small in comparison with the magnitude of the assay measurement window. This is represented by the Z’-factor, where a Z’ of 0.5 or above indicates suitability of the assay for high-throughput applications, and the maximum possible value is 1 [21]. In order to determine the Z’ value of the STAT4 assay, FP was measured from 119 wells containing 10 nM 5-CF-GpYLPQNID in the absence of protein, representing the unbound state. A further 119 wells containing 10 nM 5-CF-GpYLPQNID together with 33 nM STAT4 were measured as the bound state. Triplicate experiments resulted in an average Z’ value of 0.85 ± 0.01 (Figure 5), indicating that the assay is well-suited to high-throughput applications.

In conclusion, we have developed a fluorescence polarization-based assay for the identification of inhibitors of the STAT4 SH2 domain. The assay is stable in the presence of up to 10% DMSO and for incubation times of up to 8 h. The Z’-value of 0.85 ± 0.01 indicates that the assay is excellently suited for use in high-throughput screening campaigns, which could be used to identify new therapeutic modalities for the treatment of autoimmune diseases.

## Figures and Tables

**Table 1:** Summary of peptide inhibition results.

Peptide Sequence	Target Protein	IC_50_ [µM] or Inhibition at 40 µM [%]	K_i_ [µM] [a]
Ac-GpYLPQNID	STAT4	0.49 ± 0.04 µM	0.22 ± 0.02 µM
Ac-pYLPQTV-NH_2_	STAT3	1.27 ± 0.10 µM	0.60 ± 0.05 µM
DTpYLVLDKWL	STAT5a/5b	4.17 ± 0.13 µM	1.99 ± 0.06 µM
GpYDKPHVL	STAT1	31.4 ± 6.7 µM	15.1 ± 3.2 µM
GpYEEIP	Lck/Src	37 ± 4 % inhibition	n/a
MAGPMQSpTPLNGAKK	Plk1 PBD	2 ± 2 % inhibition [b]	n/a

[a] IC_50_ values were converted to K_i_ values using the published formula [20]. [b] n = 2. n/a: not applicable.

## Data Availability

Data are available from the corresponding author upon reasonable request.

## Supplementary Materials

The following are available online at https://www.mdpi.com/article/10.3390/mps5060093/s1, Figure S1: Binding of 5-CF-GpYLPQNID to STAT4, STAT3 and STAT6 in fluorescence polarization assays.
