# Supplementary material for: A High-Throughput Fluorescence Polarization-Based Assay for the SH2 Domain of STAT4
Source: Methods Protoc. 2022 Nov 23;5(6):93. doi: 10.3390/mps5060093 (PMC9781101; doi:10.3390/mps5060093)
Supplement: Supplementary file 1 [file mps-05-00093-s001.zip › mps-1982213-supplementary.pdf]

Figure S1

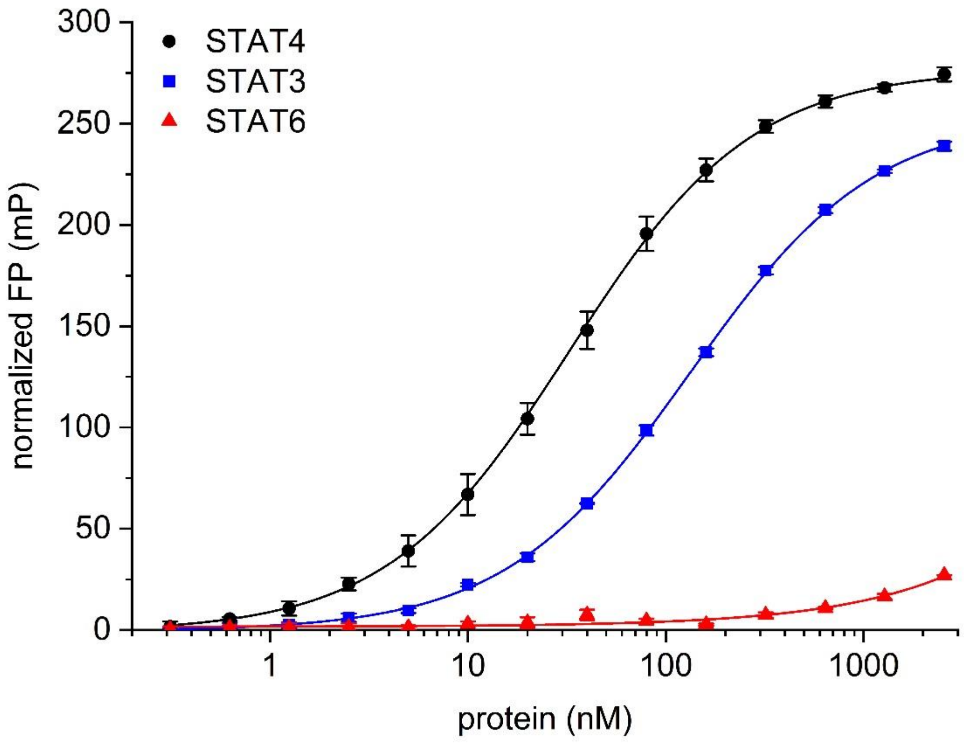

Figure S1. Binding of 5-CF-GpYLPQNID to STAT4, STAT3 and STAT6 in fluorescence polarization assays.
